# 

**Published:** 1897-06

**Authors:** 


Vol. XVIII. JUNE, 1897. No. 6.

PUBLISHED MONTHLY AT $3 A YEAR. SINGLE COPIES, 30 CENTS.

### THE JOURNAL OF COMPARATIVE MEDICINE AND VETERINARY ARCHIVES

EDITED AND PUBLISHED BY

RUSH SHIPPEN HUIDEKOPER, Veterinarian (Alfort),

W. HORACE HOSKINS, D.V.S.,

H. D. GILL, V.S.

### UNITED STATES VETERINARY MEDICAL ASSOCIATION.

**Proceedings of the Thirty-third Annual Meeting held at Buffalo, N. Y., September, 1896. (Continued.)**

Physiological Variations (*concluded*). By W. L. WILLIAMS, V.S. . . . . 193

Nail-wounds of the Foot. By S. B. NELSON, V.S. . . . . . . . 201

Feeding of Millet and Millet Disease. By T. B. HINEBAUCH, V.S. . . . . . 207

Veterinary Dentistry. By L. A. MERILLAT, V.S. . . . . . . . 219

Some Poisonous Stock Foods. By N. S. MAYO, V.S. . . . . . . . . 223

ALL COMMUNICATIONS AND PAYMENTS SHOULD BE ADDRESSED TO

**Dr. W. Horace Hoskins, Managing Editor, 3452 Ludlow St., Philadelphia, Pa.**

Copies may be had on order of all news-dealers. In London of Baillière, Tindall & Cox.

